# Optical tuning of the terahertz response of black phosphorus quantum dots: effects of weak carrier confinement

**DOI:** 10.1515/nanoph-2023-0445

**Published:** 2023-11-27

**Authors:** Xuan Liu, Lei Hou, Pengcheng Ji, Qiujin Wang, Min Wu, Yiming Xiao, Wen Xu, Lan Ding

**Affiliations:** School of Physics and Astronomy, Yunnan University, Kunming 650091, P.R. China; Key Laboratory of Materials Physics, Institute of Solid State Physics, Chinese Academy of Science, Hefei 230031, P.R. China; Micro Optical Instruments Inc., Shenzhen 518118, P.R. China; North China Research Institute of Electro-optics, Beijing 100015, P.R. China

**Keywords:** terahertz, quantum dots, black phosphorus, weak carrier confinement, two-dimensional materials

## Abstract

In contrast to few-layer black phosphorus (BP) with a relatively larger area, BP quantum dots (BP-QDs) are expected to have distinctive electromagnetic response and carrier behaviors, especially in low-frequency range such as in the THz regime. Herein, we experimentally investigate the THz properties of BP-QDs as well as the optical control of these properties. It is demonstrated that the effects of weak carrier confinement, which is associated with diffusive restoring current in each BP-QD, contribute significantly to the effective THz conductivity of BP-QDs. Instead, spectral features of discretely spaced energy levels as shown for many kinds of semiconductor QDs in UV-visible range are not observed in the THz regime. This indicates an insignificant contribution of strong quantum confinement here. Based on the modified Drude–Smith formula, we show that the optical excitation/pump of a CW laser can induce photogenerated carriers and enhance the effects of weak carrier confinement in BP-QDs. Thus, a nonlinear enhancement of THz absorption can be observed by increasing the power of the excitation laser. These results not only deepen our understanding of the fundamental physics of BP nanomaterials but also provide an alternative approach to realize active control of BP-based THz devices.

## 1 Introduction

Since 2004, the study of two-dimensional (2D) materials has become one of the most exciting areas of physics and nano-science. For example, graphene [1, 2] and 2D transition metal dichalcogenides (TMDs) [3, 4] have attracted a great of attention due to their fascinating physical features and application potential. However, for the applications in optoelectronics, monolayer graphene is subject to some limitations due to its zero bandgap [5], while TMDs suffer the drawbacks of their indirect bandgaps in multi-layer form [6]. Recently, black phosphorus (BP) in mono-layer and few-layer forms, i.e., 2D BP, is expected to compensate these shortcomings of graphene and TMDs [7–9]. This new material is a typical 2D Van der Waals crystal with a puckered hexagonal structure. In contrast to graphene and 2D TMDs, it has anisotropic optoelectronic properties and a thickness-dependent direct bandgap [10, 11]. As a result, mono-layer and few-layer BPs are extremely attractive for electronics and optoelectronics.

Although mechanical cleavage is a common technique for producing 2D BP from bulk BP [8, 12, 13], the quantity of BP nanosheets obtained by using this method is too small and the size of them is difficult to control. Therefore, pulsed laser ablation technique [14], solvothermal method [15, 16], and liquid phase exfoliation (LPE) [17–21] were used as alternative approaches to prepare BP in mono-layer and few-layer forms recently. Among them, LPE method can be used to produce 2D-BP samples with excellent stability, controllable size and thickness, as well as in high yield, including BP nanosheets (flakes) and quantum dots (QDs). For experimental research such as transport and optical measurements, the stability of 2D BPs prepared by LPE is of great significance. Nevertheless, the size of 2D BP obtained through this method is usually in the range of ∼1 nm (QD) to ∼500 nm (nanosheet) [21, 22]. This is much smaller than the available size of other 2D material that can be produced by chemical vapor deposition or epitaxial growth. Therefore, it is still a challenge to investigate the fundamental physics of 2D BPs (especially BP-QDs) based on optical measurements in low-frequency range, while it is also difficult to realize actively tuning of the optoelectronic devices based on 2D BPs. In addition, compared with BP nanosheets, BP-QDs have larger bandgaps, ultra-small sizes, and higher surface-to-volume ratios [23]. Therefore, unique physical properties and diverse applications of BP-QDs can be expected but have not been fully explored.

From the perspective of fundamental physics, in addition to transport properties, electromagnetic (optical) response is also critical to understand the features of particles and many-body interactions in 2D BPs. Recently, many experimental and theoretical works focused on the visible and infrared properties of BP nanosheets and BP-QDs [11, 14, 20–29]. In low-frequency range, such as in the terahertz (THz) regime, theoretical investigations on the properties of these samples were more common than experimental studies [30–32]. For example, surface plasmon polaritons supported by a dielectric-loaded BP waveguide were investigated [30]. A theoretical model was established to discuss the currents induced by a tilted magnetic field in 2D BP under THz radiation [32]. In contrast, previous experimental studies in THz range were mainly about the application of 2D BPs in THz photodetectors [33, 34]. In other words, up to now, experimental works focused on the basic THz response of 2D BPs (e.g., BP nanosheets and BP-QDs) and the corresponding physics are still far from sufficient, which is the prime motivation of this work. Specifically, discrete energy levels created by strong quantum confinement can be usually observed in UV-visible response of semiconductor QDs including BP-QDs [22, 27, 28], but it is still not clear that if the strong quantum confinement contributes significantly to their THz response. Furthermore, one may also be interested in the effects of other kinds of electron confinements on the THz properties of BP-QDs, e.g., weak carrier confinement.

From the perspective of device applications, the most conventional scheme to actively control the performance of devices based on 2D materials is electrically tuning [1, 4, 8, 10], in which two or three metal electrodes are usually required. However, metal electrodes not only decrease the area for light–matter interaction but also enhance the complexity of device fabrication. These shortages are especially serious for devices based on BP nanosheets or BP-QDs due to their rather small lateral sizes. In our previous works, an alternative approach without any electrodes was demonstrated, which is realized by tuning 2D-material-based devices by optical pump/excitation or optical modulation [35–38]. However, the application of this scheme in the control of electromagnetic response of 2D BPs has not been discussed in detail until now.

Based on these two aspects, in this work we investigate the THz properties of BP-QDs excited by optical irradiation. By using THz time-domain spectroscopy (THz-TDS), the THz transmission spectra of BP-QDs under laser pump/excitation were measured and then used to extract the effective optical conductivity. When the power of the pump laser increases, a nonlinear enhancement of THz absorption arises. However, spectral features of discretely spaced energy levels are not observed here, indicating an insignificant contribution of strong quantum confinement. More importantly, we demonstrate that the frequency-dependent and optically-tunable behaviors of the effective optical conductivity can be explained well by the modified Drude–Smith formula, which includes the contribution of weak carrier confinement induced by diffusive restoring current in each BP-QDs. Based on the formula, we not only extract four significant characteristic parameters of the BP-QDs but also show the optical tunability of them. These results can deepen our understanding of the fundamental properties of BP-based nanomaterials and find applications in tunable THz devices.

## 2 Results and discussion

### 2.1 Sample preparation and characterization

In brief, by using N-methyl-2-pyrrolidone (NMP) as solvent for LPE, we produced large quantities of BP-QDs. The concentration of the NMP dispersion of BP-QDs was about 0.1 mg/mL. Then, 0.15 mL of the dispersion was dropped onto a sapphire substrate. After spin coating and drying in a vacuum dryer, the sample of BQ-QDs was obtained. The details of our preparation process can be found in Appendix A. It should be noted that another bare sapphire substrate was used as the reference in our THz measurements. The thicknesses of the substrate under the BP-QDs and of the reference (bare) substrate are defined as *d*_sam_ and *d*_ref_, respectively. These two substrates were cut apart from the same sapphire plate, which is highly plane parallel and possesses very flat surfaces. The thicknesses of these two substrates were experimentally determined as *d*_sam_ = *d*_ref_ = 497 μm. Accordingly, an error in the relative phase change of the THz wave transmitted through the BP-QDs, which will be introduced by the difference between *d*_sam_ and *d*_ref_, can be neglected.

**Figure 1:**
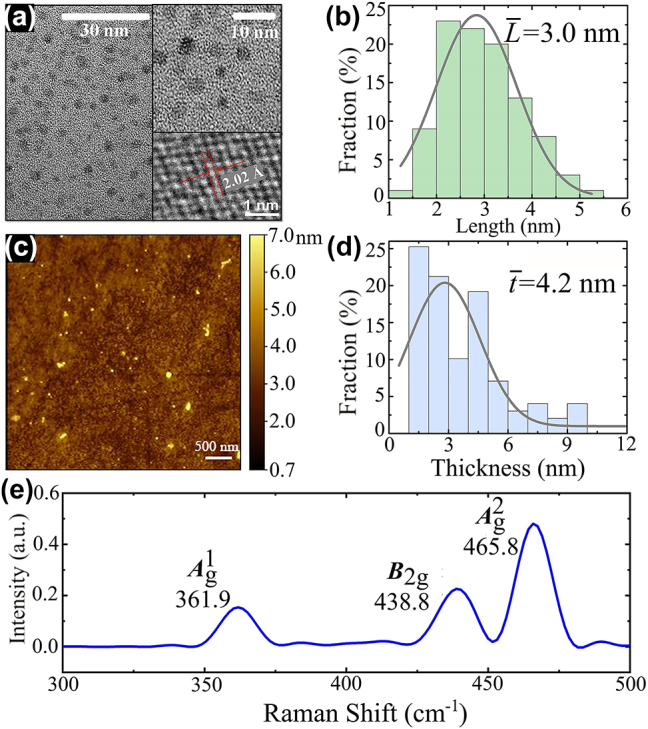
Basic characterization. (a) TEM and (c) AFM images of the BP-QDs and the corresponding statistical analysis of (b) the lateral length and (d) thickness. (e) Raman spectrum of the BP-QDs. The inset to (a) shows the HR-TEM image of lattice fringes.

Next, to characterize the basic properties and quality of the sample, transmission electron microscopy (TEM), atomic force microscopy (AFM), and Raman spectroscopy were performed. The morphology and crystallinity of the BP-QDs are shown in Figure 1(a). Most of the BP-QDs are non-percolated and well-distributed on the substrate. Furthermore, clear lattice fringes can be observed from the high-resolution TEM (HR-TEM) image. For example, the spacing of 2.02 Å corresponds to (022) plane of a BP crystal, which is consistent with the BP lattice parameter [39]. The uniform lattices suggest that BP in the form of QDs retains the original crystalline state. Figure 1(c) shows the AFM image of a larger area of the BP-QD sample. Based on the statistical TEM and AFM analysis of quantitative BP-QDs, i.e., Figure 1(b) and (d), the average length and average thickness are obtained as ∼3.0 nm and ∼4.2 nm, respectively.

Although AFM can measure the lateral lengths (*L*) and thicknesses (*t*) of nanoparticles simultaneously, two limitations should be noted. (i) Due to the finite size of AFM tips, the lateral lengths of nanoparticles would be usually overestimated by AFM [27]. Thus, we utilized the AFM image only for thickness analysis. (ii) Since the resolution of the AFM used here is lower than that of the TEM, some of the BP-QDs cannot be well resolved and thereby the thickness statistical analysis is not as precise as the length statistical analysis. In addition, one can find from the AFM image that the agglomerations of a small amount of BP-QDs (less than 5 %) result in false particle signals with lengths of 30∼60 nm. Nevertheless, we can hardly find particle signals for *L* = 7–30 nm in both of the TEM and AFM images. Therefore, these signals (7–60 nm) are not included in our statistical analysis for the BP-QDs. The QD agglomerations can respond the THz wave, but this contribution is much weaker than that of a large number of isolated BP-QDs. Further evidence and discussion can be found in Section 2.2.

Moreover, three characteristic peaks of the BP-QDs can be detected by Raman spectroscopy, as illustrated in Figure 1(e). The peak located at 361.9 cm^−1^ can be ascribed to out-of-plane phonon modes ( $A_{\text{g}}^{1}$ ), whereas the other two peaks at 438.8 and 465.8 cm^−1^ are attributed to in-plane phonon modes (*B*_2g_ and $A_{\text{g}}^{2}$ ), respectively. These peaks correspond to three of the characteristic Raman peaks of bulk BP crystal but blue-shift slightly. It demonstrates that the BP-QDs still exhibit the unique puckered hexagonal layered structure but possess few layers [14, 21]. In addition, the integrated intensity ratio of $A_{\text{g}}^{1}/A_{\text{g}}^{2}$ can be kept larger than 0.3 for a long time, indicating that the degeneration/oxidation level of our sample is very low and the BP-QDs obtained by LPE are stable enough [13]. This is because the solvation shell acts as a barrier to prevent oxidative species reaching the BP-QD surface and edge [20].

### 2.2 Optically tunable THz response of BP-QDs

**Figure 2:**
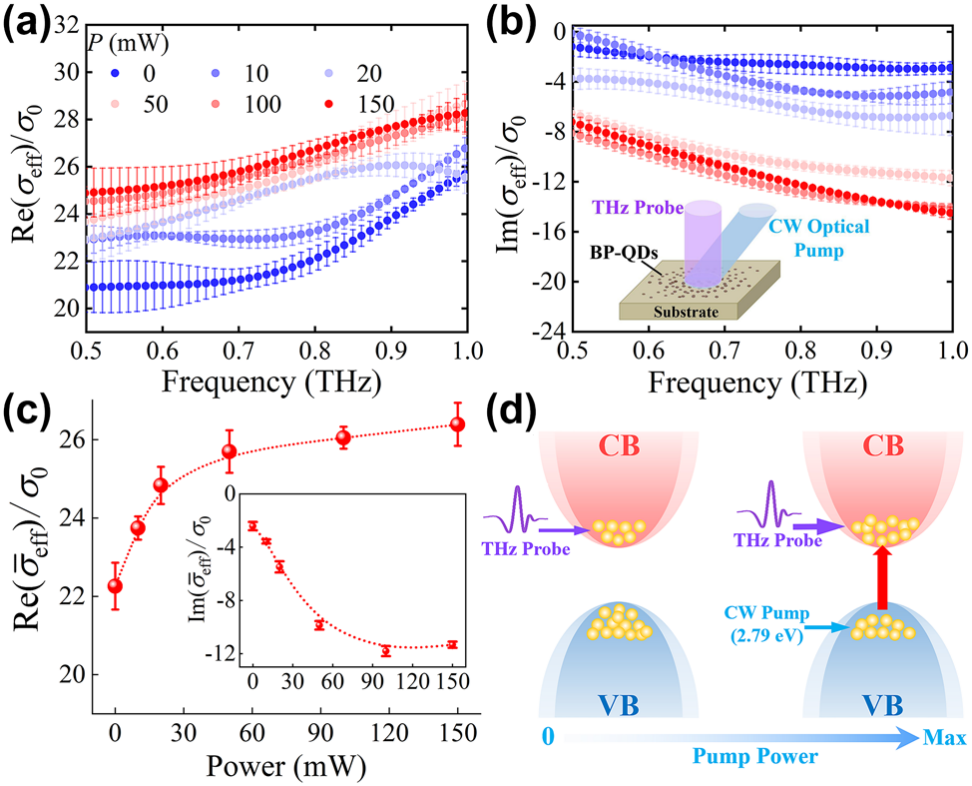
THz response of BP-QDs under laser excitation. The (a) real and (b) imaginary parts of the effective optical conductivity of the BP-QDs. Note that the THz frequency is defined as *f* = *ω*/2π and *σ*_eff_(*ω*) is normalized by the universal conductivity *σ*_0_ = *e* ^2^/4*ℏ*. The inset to (b) shows the schematic of the experimental geometry for CW optical pump-THz probe measurements. (c) The real part of the frequency-average value ${\bar{\sigma }}_{\text{eff}}$ as a function of the pump power. The imaginary part of ${\bar{\sigma }}_{\text{eff}}$ is also presented in the inset of (c). Error bars in these figures correspond to the standard deviation of the average value obtained from data accumulations within multiple measurements. (d) Schematic band diagrams under weak and strong laser excitation. The valence band and conduction band of the BP-QDs are denoted by VB and CB, respectively. The carriers (electrons) associated with the THz absorption are illustrated as yellow balls. We use blue and red arrows to represent the laser absorbed by the BP-QDs and the corresponding photon-induced inter-band transition, respectively. The thin/thick purple arrow represents the weak/strong THz absorption by carriers.

Based on a THz-TDS system in conjunction with a continuous-wave (CW) laser, we measured the THz response of BP-QDs excited by visible light (445 nm), as shown in the inset of Figure 2(b). Furthermore, since the THz response could not be strong due to the small amount of BP, we should ensure that the laser-induced change of the THz response and the spectral noise can be well distinguished. Accordingly, we only discuss the THz properties within the effective bandwidth [40] (0.5–1.0 THz). Details can be found in Figure 7(a) in Appendix B. To improve the reliability of the measured data, the CW optical pump-THz probe experiment was carefully performed for three times.

At a given pump power *P*, the THz pulses transmitted through the sample (BP-QDs/sapphire) and through the bare sapphire substrate were measured as the sample and reference signals, respectively. By the Fourier transformation of these two time-domain signals, we can derive the complex electric fields in frequency-domain for the sample and reference, i.e., *E*_sam_(*ω*) and *E*_ref_(*ω*), respectively. Therefore, the relative THz transmission through the BP-QDs can be obtained as *T*(*ω*) = *E*_sam_(*ω*)/*E*_ref_(*ω*), where *E*_ref_(*ω*) is found to be nearly independent to *P*. Then, we calculate the relative amplitude |*T*(*ω*)| and phase change arg[*T*(*ω*)] for the BP-QDs excited by different laser power (see Figure 8 in Appendix B). As mentioned in Section 2.1, we can safely neglect the error in arg[*T*(*ω*)] introduced by the difference between *d*_sam_ and *d*_ref_.

Because the THz wavelength is much larger than the average lateral length of the BP-QDs and the average spacing between them, the THz wave cannot resolve the details of the BP-QDs and sees only an average THz response [41, 42]. This response can be described by an effective optical conductivity *σ*_eff_(*ω*). It should be noted that *σ*_eff_(*ω*) characterizes not only the intrinsic response of BP inside the QDs but also the effects of finite size of these nanoparticles. Based on *T*(*ω*), we can extract the effective optical conductivity through the Tinkham relation [43]:

(1) $$\sigma_{\text{eff}}\left(\omega \right)=\frac{1+\tilde{n}}{T\left(\omega \right)Z_{0}}-\frac{1+\tilde{n}}{Z_{0}}$$

in which $\tilde{n}=3.05$ is the refractive index of the sapphire substrate measured by our THz-TDS system (see Figure 7(b) in Appendix B), *Z*_0_ = 377 Ω is the impedance of the free space.

For a thin film pumped/excited by visible light, multiple reflections of the light in this film can enhance the photoexciting effect. Thus, the THz response of a photoexcited film may be overestimated [44]. Based on this effect, the Tinkham approximation may lead to inaccurate THz conductivity of the film. In fact, this situation will be definitely encountered if the wavelength of the excitation/pump light is comparable to (or smaller than) the film thickness and the optical penetration length. In contrast, the Tinkham approximation is only valid when the optically excited layer fulfill *k*_0_ *t*_p_ *n*_p_ ≪ 1, where *k*_0_ = *ω*/*c* is the wave vector of vacuum, *t*_p_ is the thickness of the photoexcited layer in a film, and *n*_p_ is the complex refractive index of the photoexcited layer. In our experiment, the THz response of the BP-QDs can be approximately equivalent to that of an effective film with a thickness of about $\bar{t}=4.2\;\;\text{nm}$ and an optical conductivity of *σ*_eff_(*ω*). Since the thickness is much smaller than the wavelengths of the pump laser (445 nm) and the THz wave (300–600 μm), the multiple reflections of the laser and the THz wave can be neglected. Furthermore, the pump laser can definitely penetrate the effective film of BP-QDs, leading to $t_{\text{p}}=\bar{t}$ . By considering the fact that *n*_p_ is impossible to be very large, we can safely obtain *k*_0_ *t*_p_ *n*_p_ = 10^−5^ × (4.4 ∼ 4.8)*n*_p_ ≪ 1. Therefore, the effective optical conductivity of the BP-QDs can be extracted accurately by using the Tinkham relation of Equation (1).

Although we have verified the condition of *d*_sam_ = *d*_ref_, it is known that an error in the experimental determination of *d*_sam_ may also lead to an uncertainty in the optical parameter of the BP-QDs [45]. We denote this error by Δ*d*_sam_ here. Specifically, since the substrate is nearly lossless (*κ* = 0) within the frequency band of research, Δ*d*_sam_ can only result in an uncertainty Δ*n* in the real part of the complex refractive index ( $\tilde{n}$ ) of the substrate. Thus, an error (Δ*σ*_eff_) will be introduced in *σ*_eff_(*ω*) due to the uncertainty of $\tilde{n}$ in Equation (1). Based on the given substrate parameters, it is easy to roughly estimate the accuracy of *σ*_eff_(*ω*). For example, if we assume a case of Δ*d*_sam_ = ±10 μm (i.e., *d*_sam_ = *d*_ref_ = 497 ± 10 μm), which is a rather large error and not common in THz experiments, Δ*n* is calculated as about ±0.04 and thereby the ratio between Δ*σ*_eff_ and *σ*_eff_(*ω*) can be obtained as only about 1 %. In fact, the realistic situation is |Δ*d*_sam_|<<10 μm. It means that *σ*_eff_(*ω*) is almost robust against the error in the determination of *d*_sam_.

As shown in Figure 2(a) and (b), the measured data is mainly expressed by the average values and their errors for clarity. The error bars correspond to the standard deviation of the average value obtained from data accumulations within multiple measurements (three times of measurements), indicating the measurement accuracy. Interestingly, we find no spectral features of discrete energy levels in *σ*_eff_(*ω*), which means that the strong quantum confinement hardly affects the THz response of BP-QDs. This is very different from the situations of the UV-visible measurements of BP-QDs [22, 27, 28]. According to the overall trend, one can see from the figures that Re(*σ*_eff_) and Im(*σ*_eff_) enhance with increasing *P* at most of the frequencies. Moreover, Im(*σ*_eff_) is apparently smaller than Re(*σ*_eff_) at a given power, which means that the BP-QDs affect the amplitude of the transmitted THz wave more significantly than affect the phase change of it.

By comparing the THz response of the BP-QDs with the results obtained in some previous studies on other nanoparticles [46–48], we find some differences in the shape and magnitude of the optical conductivity spectra. Because the form, bandgap, and distribution of our BP-QD sample is different from those of the nanoparticles reported in the aforementioned works, these differences are entirely possible and reasonable. For example, the optical conductivity spectra of our sample are relatively smooth and have no oscillations, which can be attributed to the absence of the multiple reflections of THz wave due to the ultra-small *t*. Obviously, *t* is much thinner than the plates compressed with nanoparticle powders and the cuvettes containing nanoparticle dispersion [46, 47], so the thickness of the effective BP-QD layer is deep-subwavelength and thereby the multiple reflections of THz wave cannot occur. Furthermore, comparing with HgTe nanocrystals [48], the bandgap of BP is much larger and the BP-QD distribution is not highly dense. Therefore, the spectral contribution of a few QD agglomerations (30–60 nm) will not lead to significantly enhanced THz response via larger particle size. In other words, the THz response of these agglomerations could not dominate over that of the real BP-QDs.

From the view of overall behavior of the THz response, our results are consistent with the previous studies on other nanoparticles. One can see that the real part of *σ*_eff_(*ω*) does not decrease monotonically with increasing *ω*, while the imaginary part is negative. These are typical evidences of weak carrier confinement rather than features of intra-band-like transport of free carriers. The effect of weak carrier confinement in nanoparticles or QDs can be well described by the modified Drude–Smith model [47, 49, 50], which presents the blue shift of the plasmonic peak at *ω* = 0 in the Drude formula [30]. In short, the features of *σ*_eff_(*ω*) are akin to the THz response of other kinds of nanoparticles, such as non-percolated semiconduction nanocrystals [47]. It should be noted again that the strong quantum confinement in nanoparticles (including our BP-QDs) may give rise to discrete quantum levels, but the spectral features of these discrete levels have been hardly observed in the THz regime. A theoretical model that describes the effective optical conductivity will be discussed in detail in Section 2.3.

Next, one can see from Figure 2(a) and (b) that *σ*_eff_(*ω*) can be effectively modulated by the laser. By calculating the integral of *σ*_eff_ in the frequency range of 0.5–1.0 THz, the pump power dependence of the frequency-average value of *σ*_eff_ is depicted in Figure 2(c). Note that the frequency-average value ${\bar{\sigma }}_{\text{eff}}$ can be expressed as ${\bar{\sigma }}_{\text{eff}}=\int_{\omega }\sigma_{\text{eff}}\left(\omega \right)d\omega /\Delta \omega$ with Δ*ω* being the frequency bandwidth. We can observe nonlinear increase of $\text{Re}\left({\bar{\sigma }}_{\text{eff}}\right)$ and $\text{Im}\left({\bar{\sigma }}_{\text{eff}}\right)$ with *P*. It is well known that the absorption coefficient is proportional to the real part of optical conductivity. Therefore, the result of $\text{Re}\left({\bar{\sigma }}_{\text{eff}}\right)$ demonstrates that the THz absorption of the BP-QDs can be modulated/tuned by purely optical means instead of electric approach. In the power range of 0–150 mW, the modulation depth of $\text{Re}\left({\bar{\sigma }}_{\text{eff}}\right)$ or THz absorption can reach 24 % at most.

The interpretation of this phenomenon is illustrated in Figure 2(d). Note that the conventional band diagram of BP is used here for brief and approximate discussion, because there are no spectral features of discrete levels induced by strong quantum confinement in our results. The physical mechanism can be summarized as follows: (i) Since the photon energy of THz wave is undoubtedly below the band gap of BP (0.3–1.7 eV) [24, 26], only intra-band transition of carriers can be induced by the absorption of THz probe pulse. (ii) When the pump/excitation laser with photon energy of 2.79 eV irradiates the BP-QDs, photogenerated carriers emerge in large numbers via the process of inter-band transition. (iii) The photogenerated carriers can absorb more THz photons, resulting in the increase of $\text{Re}\left({\bar{\sigma }}_{\text{eff}}\right)$ . (iv) Due to the limited number of electrons in the valence band as well as the effects of the laser on the characteristic properties of carriers, the THz absorption associated with the photogenerated carriers nonlinearly increases with *P* and then gradually saturates. The microscopic origin of this phenomenon will be discussed in detail later. Obviously, the performance of THz absorption and the optical modulation of it can be further improved by preparing samples via high-concentration dispersion of BP-QDs, which is flexible and low-cost. Moreover, the method of purely optical modulation can overcome the shortages of electrically tuning and find applications in all-optical THz devices based on 2D BPs.

### 2.3 Mechanism and characteristic parameters

In previous works, the THz response of 2D BP (nanosheet or large-size sheet) was usually described by the Drude formula [30], which is based on free-electron-gas approximation. In this formula, the real part of optical conductivity should decrease monotonically with increasing *ω*, while the imaginary part should be positive. However, most of the experimental data shown in Figure 2(a) and (b) does not obey this law, so the conventional Drude model cannot describe the THz response of BP-QDs rightly. Meanwhile, because no sharp rising edges or peaks are observed in the spectra of Re(*σ*_eff_), the absorption of phonon/exciton/plasmon resonances has no contribution in the range of 0.5–1.0 THz. Therefore, the Drude-Lorentz formula including Lorentzian terms is also not suitable here. In essence, since our sample is a typical nanomaterial consisting of nanostructures (i.e., QDs), the weak carrier confinement induced by diffusive restoring current should be considered [49], which leads to the modified Drude–Smith formula:

(2) $$\sigma_{\text{eff}}\left(\omega \right)=\frac{\omega_{\text{p}}^{2}\varepsilon_{0}\tau^{\prime }}{1-i\omega \tau^{\prime }}\left(1-\frac{C}{1-i\omega t_{\text{diff}}}\right)$$

in which *τ*′ is the intrinsic electronic scattering time, *t*_diff_ is the diffusion time of carriers, and $\omega_{\text{p}}=\sqrt{Ne^{2}/\varepsilon_{0}m^{*}}$ is the plasma frequency with *N* and m* being the carrier density and effective electron mass, respectively. The factor *C* = [0, 1] is used to describe the contribution of weakly confined charge carriers. This parameter is dependent on the size of a single nanostructure and its boundary reflectivity. In our sample, most of the quantum dots are distributed and isolated, so the boundary reflectivity should be 100 %. Thus, here *C* is mainly decided by the average size of the BP-QDs, especially the average lateral size. Moreover, as mentioned before, all these parameters characterize the average/effective response of the BP-QDs located in the area of THz illumination rather than the response of a single QD.

**Figure 3:**
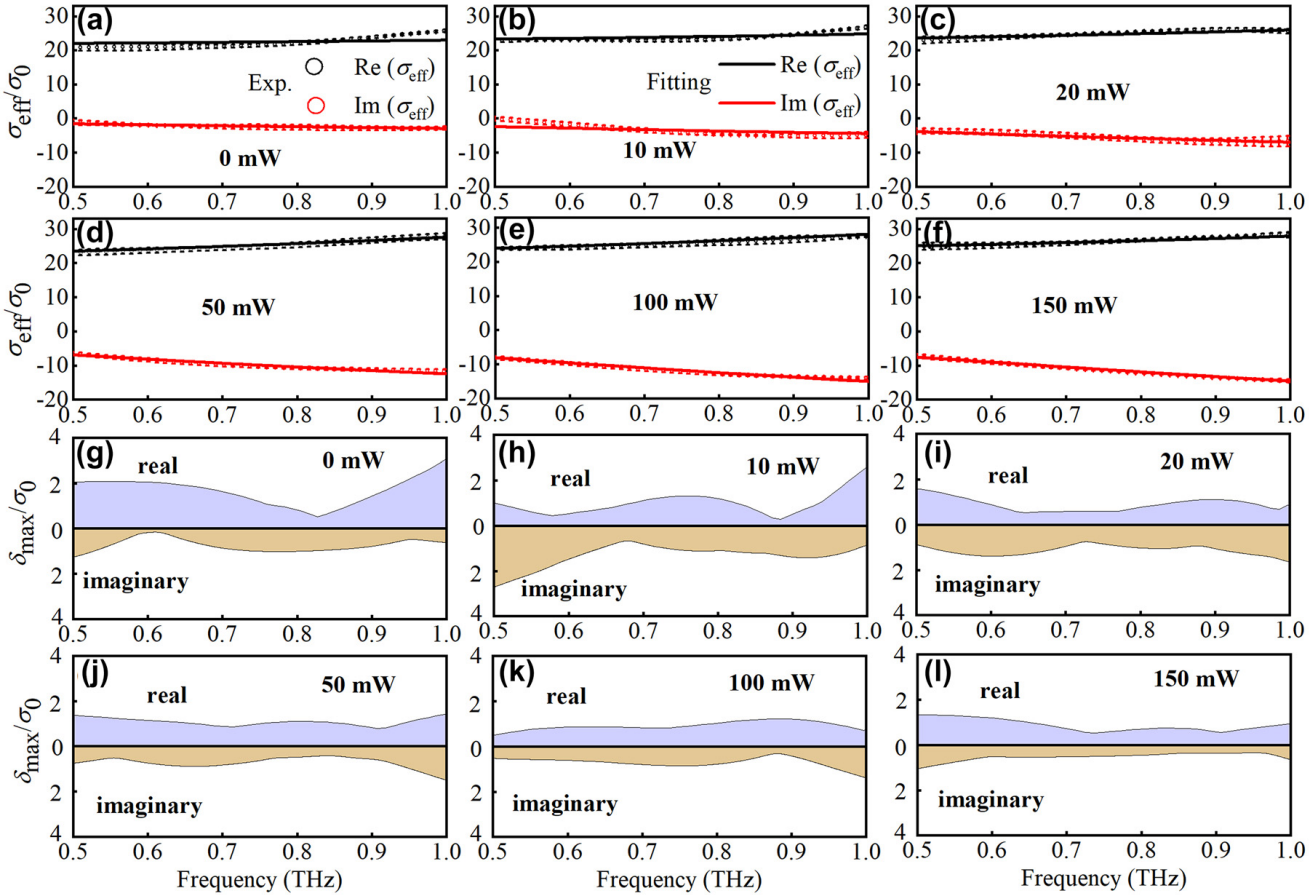
Comparison of the measured and fitted results. The measured effective optical conductivity (average value) is fitted by the modified Drude-Smith formula for (a) *P* = 0, (b) 10 mW, (c) 20 mW, (d) 50 mW, (e) 100 mW, and (f) 150 mW. Error bars show the standard deviation of the average value obtained from data accumulations within multiple measurements. To clearly illustrate the stability of the fitting, the modulus of the maximum deviation between the fitting curve and the experimental data is provided for (g) *P* = 0, (h) 10 mW, (i) 20 mW, (j) 50 mW, (k) 100 mW, and (l) 150 mW.

Figure 3(a)–(f) show the comparison between the experimental results and the fitting ones. We provide the fitting data as well as the experimental data with error bars. The fitting data is obtained by reproducing the average value of *σ*_eff_(*ω*) calculated from data accumulations within multiple measurements. To show the stability of the fitting process, we also present the modulus of the maximum deviation (i.e., *δ*_max_) between the fitting curve and the average value of measured data in Figure 3(g) to (l). Most of the experimental data can be reproduced well by Equation (2). However, for lower pump power such as *P* ≤ 10 mW, one can find that the model deviates from the experimental data relatively obviously. This discrepancy can be attributed to two reasons: (i) The effective carrier density *N* is relatively small, leading to weak response of carriers. (ii) For weak laser excitation, the average lateral length $\bar{L}$ may approach (or even smaller than) the carrier mean free path *l* ≈ *v*_th_ *τ*′ or the carrier diffusion length $Z_{\text{diff}}\approx v_{\text{th}}\sqrt{t_{\text{diff}}\tau^{\prime }}\geq l$ , where $v_{\text{th}}=\sqrt{k_{\text{B}}T/m^{*}}$ is the thermal velocity [49, 50]. In this situation, the effect of strong quantum confinement may affect the carrier behaviors by a complex way, which should be discussed in a quantum mechanical system rather than a classical model such as Equation (2).

As the laser power increases, the response of carriers enhances with increasing *N*, while the limitation of $\bar{L}>Z_{\text{diff}}>l$ can be fully satisfied due to the decrease of the mean free path and diffusion length. Obviously, *δ*_max_ tends to decrease with increasing *P*, also indicating more significant effect of weak carrier confinement for relatively strong excitation. Therefore, Figure 3(c)–(f) shows excellent agreement between the experimental and fitting data. Furthermore, we can roughly estimate the boundary values of *l*, *Z*_diff_, and m* in this experiment based on the aforementioned discussion.

**Figure 4:**
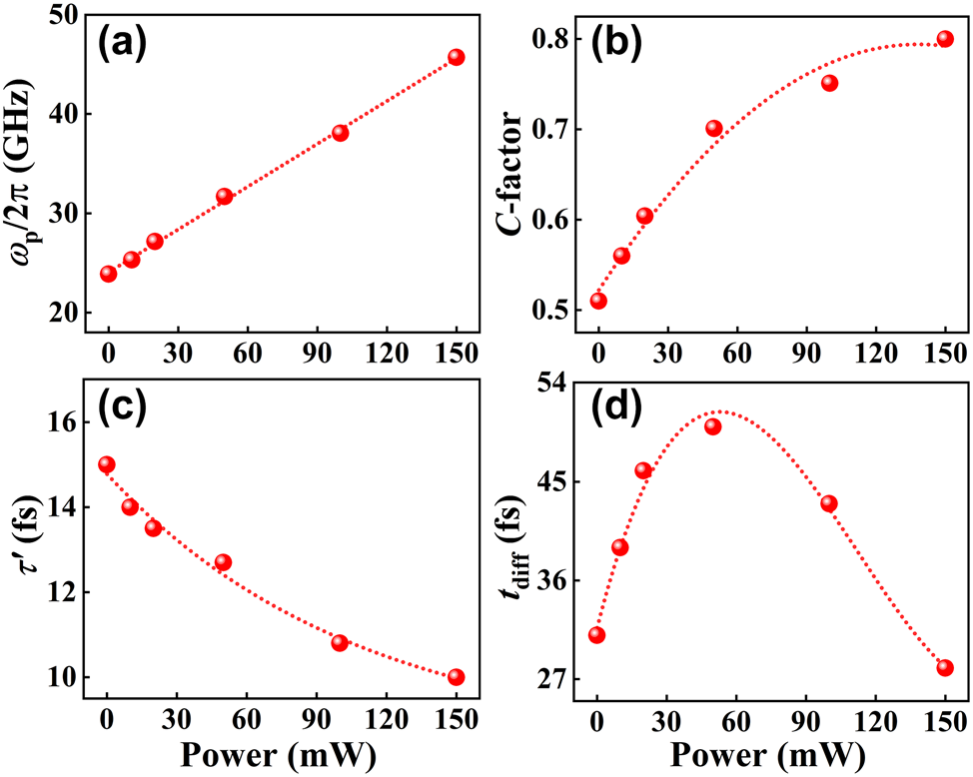
Characteristic parameters. The effects of the pump power on the (a) effective plasma frequency, (b) confinement factor, (c) intrinsic scattering time, and (d) diffusion time. The dotted curves are drawn to fit the experimental data or guide the eye.

Through the fitting process, the effective characteristic parameters as functions of the pump/excitation power are extracted, as illustrated in Figure 4. Based on the stability analysis of the fitting via *δ*_max_, we believe that the characteristic parameters extracted from the average value of *σ*_eff_(*ω*) are representative and reliable. In Figure 4(a), the quasi-linear increase of *ω*_p_ with *P* can be understood by the mechanism shown in Figure 2(d). Stronger laser illumination can excite more photogenerated carriers, which results in the increasement of the plasma frequency, i.e., $\omega_{\text{p}}\propto \sqrt{N}\propto P$ . Thus, the contribution of the Drude term in Equation (2) can be enhanced, as mentioned previously. It is also known that the THz absorption is usually proportional to the carrier density *N* in a semiconductor, so one may expect the relation of $\text{Re}\left({\bar{\sigma }}_{\text{eff}}\right)\propto N\propto P^{2}$ for the BP-QDs. However, we haven’t observed this kind of quadratic relation between $\text{Re}\left({\bar{\sigma }}_{\text{eff}}\right)$ and *P* in Figure 2(c). The strong effects of the pump light on the kinematic characteristic parameters of carriers (e.g., *t*_diff_ and *τ*′) can mainly give rise to this discrepancy.

Figure 4(b) illustrates that the confinement factor *C* increases nonlinearly with *P* and then saturates at strong laser excitation. The saturation value is about *C* = 0.8 instead of 1. For relatively larger nano-particles, e.g., $\bar{L}∼100\;\;\text{nm}$ [50], the boundary reflectivity of 100 % usually results in *C* = 1. This difference can be attributed to the effect of small particle size. As mentioned previously, *C* depends on the boundary reflectivity and lateral size of nanostructure, whose accurate functional relation and the corresponding mechanism are not clear until now [49]. Therefore, further investigations on this topic are interesting and necessary but beyond this work.

One can see from Figure 4(c) and (d) that the intrinsic scattering time and diffusion time can be nonlinearly tuned by *P*. Specifically, the intrinsic scattering time decays exponentially as $\tau^{\prime }=\tau_{1}\exp\left(-P/P_{0}\right)+\tau_{0}$ , in which *P*_0_ = 108.2 mW, *τ*_1_ = 6.4 fs, and *τ*_0_ = 8.4 fs. The decrease of *τ*′ is associated with the enhanced probability (or rate) of electron scattering for increased carrier density, but the enhancement of the scattering probability (or rate) may gradually saturate for high carrier density. Therefore, the scattering time remains around 8.4 fs when the laser power exceeds 150 mW.

**Figure 5:**
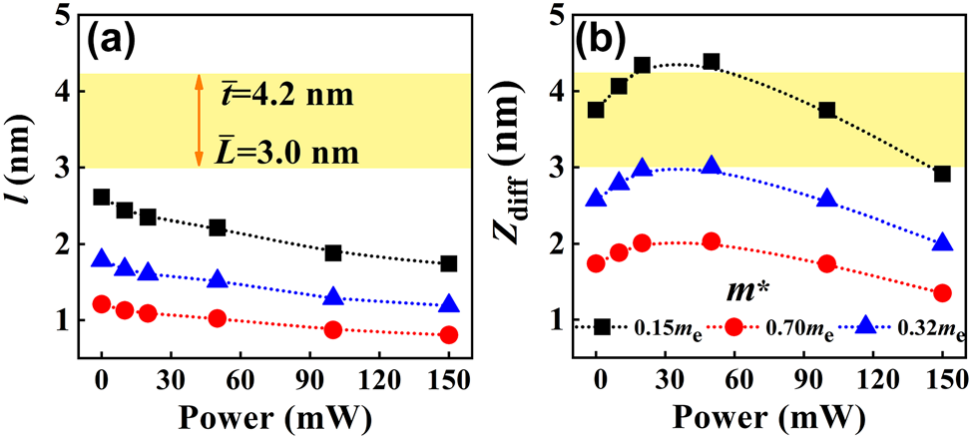
Estimated characteristic lengths. The power-dependent (a) mean free path and (b) diffusion length. The yellow region denotes the average sizes of the BP-QDs, whereas the dotted curves are drawn to guide the eye.

**Figure 7:**
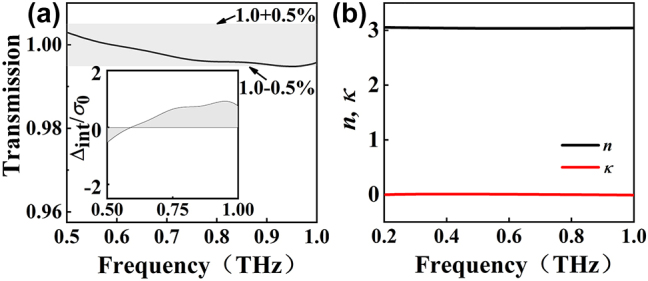
Intrinsic error and substrate response. (a) Determination of the effective bandwidth and (b) the complex refractive index of the sapphire substrate. The inset of (a) provides the translated error in Re(*σ*_eff_) calculated based on the transmission error less than |±0.5 %|.

**Figure 8:**
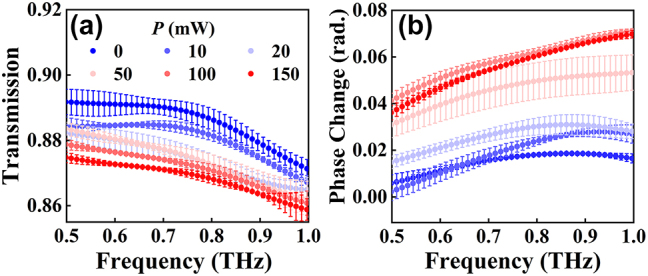
Relative THz transmission through BP-QDs under laser excitation. (a) The excitation power-dependent relative amplitude and (b) phase change of *T*(*ω*). Error bars show the standard deviation of the average value obtained from multiple measurements.

In contrast, the diffusion time *t*_diff_ does not change monotonically with the pump power but can be fitted by a third order polynomial of *P*. It reaches a maximum of 51.4 fs at about 53.0 mW. This behavior is a result of the competition between the increase of *N* and the decrease of *τ*′. Moreover, to ensure the validity of our model, we can estimate the boundary values of *l*, *Z*_diff_, and m* based on *t*_diff_ and *τ*′. Under the limitation of $\bar{L}>Z_{\text{diff}}>l$ , one can derive the lower boundary of the effective electron mass $m_{\text{b}}^{*}$ of our BP-QDs:

(3) $$m^{*}\geq m_{\text{b}}^{*}\approx \frac{t_{\text{diff}}\tau^{\prime }k_{\text{B}}T}{{\bar{L}}^{2}}=0.32m_{\text{e}}$$

in which the product of *t*_diff_ *τ*′ should be its maximum. Equation (3) means that if m* is smaller than $m_{\text{b}}^{*}$ , the quantum effects cannot be neglected in the BP-QDs and thereby the fitting performance of Equation (2) may become very poor. Figure 5 shows the values of *l* and *Z*_diff_ for different m*, in which m* = 0.15*m*_e_ and m* = 0.7*m*_e_ correspond to the effective electron masses in the AC-direction and ZZ-direction for monolayer BP [30], respectively. It should be emphasized that the discrepancy between the experimental and fitting data for smaller *P* is not only associated with $\bar{L}<Z_{\text{diff}}$ or $\bar{L}<l$ but also related to the low carrier density.

## 3 Conclusions

In conclusion, by using THz-TDS we investigate the carrier properties of BP-QDs under CW-laser excitation. The effective optical conductivity of the BP-QDs as well as its laser power-dependence are obtained. It is demonstrated that the THz absorption of the BP-QDs can be modulated/tuned by changing the power of the CW laser. This property can provide an alternative approach to realize active control of BP-based devices such as filters, modulators, and absorbers.

From the microscopic view, we find that the weak carrier confinement induced by diffusive restoring current can result in the distinctive THz response of BP-QDs. Moreover, the effects of excitation power on the characteristic parameters (e.g., *ω*_p_, *τ*′, *t*_diff_, and *C*) are discussed and explained. Based on these parameters, we also roughly estimate the mean free path and diffusion length, and then obtain the lower boundary of the effective electron mass as m* ≥ 0.32*m*_e_. These results are helpful to gain an in-depth understanding of the carrier properties of BP nanomaterials.

## Figures and Tables

**Figure 6:**
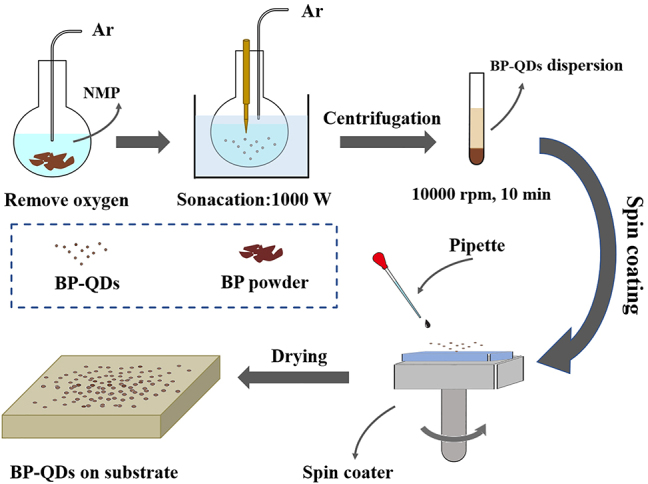
Schematic illustration of the preparation process of BP-QDs sample.

## Appendix A: Details of the sample preparation and basic characterization

The preparation process of the BP-QD sample is shown in Figure 6. Black phosphorus powder (15 mg) was added to NMP solvent (100 mL). We bubbled the mixture with argon (Ar) for 10 min to remove the dissolved oxygen. Under cooling and Ar protection, the mixture was sonicated for 5 h at more than 50 Hz with a horn-probe sonic tip (2 kW) to conduct the liquid exfoliation of bulk BP, yielding a stock dispersion. Next, the dispersion was centrifuged at 10,000 rpm for about 10 min to remove any non-exfoliated bulk BP and relatively thick/large BP nanosheets. The supernatant containing BP-QDs was collected and configured to the required concentration (e.g., 0.1 mg/mL). Then, we dropped 0.15 mL of the BP-QD dispersion onto a sapphire substrate. To prepare uniformly dispersed sample, the BP-QD dispersion on the sapphire was spin coated by two steps (500 rmp for 12 s followed by 3000 rpm for 60 s). After drying in a vacuum dryer, we obtained the BP-QDs with an area of about 0.8 cm × 0.8 cm on the substrate.

At room temperature (300 K), the morphology, size distribution, and thickness distribution of the sample were characterized by TEM and AFM technology. The size distribution and thickness distribution of the BP-QDs were determined based on the TEM and AFM images obtained by a TEM system (Tecnai G2 F20) and an AFM system (SPI-3800), respectively. By using high-resolution TEM (JEM-2100), we can observe the lattice structure of BP-QD. The Raman spectrum of the BP-QDs was recorded by using a standard setup of Raman spectrometer (RENISHAW inVia) with an excitation wavelength of 532 nm.

## Appendix B: THz setup, effective bandwidth, and transmission measurements

The experimental setup for CW optical pump-THz probe measurements was established based on a standard THz-TDS system. The femtosecond laser with a central wavelength of 800 nm and a pulse duration of 35 fs was generated by a Ti: sapphire amplifier (Coherent, Astrella) with a repetition rate of 1 kHz. This laser beam was split into a pump beam and a probe beam for THz generation and detection, respectively. We controlled the time delay between the pump and probe pulses by dual retro-reflectors driven by a servo. Based on a LiNbO_3_ crystal, the pulsed THz wave was generated by using a tilted-pulse-front technique. To avoid nonlinear THz effects in the sample, we limited the peak electric field below ∼7 kV/cm by fixing the power of the pump beam as ∼100 mW. Then, the THz wave transmitted through the sample (or reference material) and irradiated on a ZnTe crystal together with the probe laser beam. Based on a standard electro-optic sampling technique, the transmitted THz wave was detected. The working bandwidth of this system was ∼1.9 THz. Moreover, we introduced a CW optical beam with a fixed wavelength (445 nm) and tunable power (0–150 mW) to excite/pump the sample. This beam was generated by a solid-state CW laser and illuminated the sample surface at an incident angle of 45°. The THz wave and the CW optical beam were overlapped on the sample with spot sizes of ∼1.5 and ∼5 mm, respectively. Note that the THz measurements were performed at room temperature (300 K).

To improve the reliability of the measured data, we carefully performed the CW optical pump-THz probe experiment for three times. All measurements were conducted in a dry nitrogen atmosphere. The temperature near the sample holder was fixed by a thermostat and real-time monitored. The THz scan length in time domain was about 90 ps and the corresponding frequency resolution was about 0.011 THz, which can present the spectral details well enough.

Moreover, we examined the relative THz transmission amplitude based on two consecutive measurements without reference substrate or sample. The relative amplitude deviates less than 0.5 % away from 100 % in the frequency range of 0.5–1.0 THz. This range can be defined as the effective bandwidth of this experiment [40], as shown in Figure 7(a). In fact, we mainly use the transmission deviation to describe the spectral noise of the THz system. The value of 0.5 % is an experience value in measuring 2D materials rather than a very strict boundary. By using Equation (1), we translate this transmission error (less than |±0.5 %|) to the intrinsic error in Re(*σ*_eff_). The result can be obtained as about |Δ_int_|≤0.9*σ*_0_ and shown in the inset of Figure 7(a). Obviously, the intrinsic error Δ_int_ is small enough and thereby affects the laser-induced change of the THz response very weakly. Furthermore, it should also be noted that this error has been included in the data errors of multiple measurements. Thus, it is no need to add Δ_int_ in the data errors in Figure 2(a)–(c), Figure 3(a)–(f), and Figure 8 again.

We also measured the complex index ( $\tilde{n}=n+i\kappa$ ) of the sapphire substrate used in this work, as illustrated in Figure 7(b). In THz range, the substrate is lossless (*κ* = 0). Meanwhile, we verify that the complex index of the substrate is nearly independent on the power change of the excitation laser. Next, based on the relative THz transmission through the BP-QDs, i.e., *T*(*ω*) = *E*_sam_(*ω*)/*E*_ref_(*ω*), one can calculate the relative amplitude |*T*(*ω*)| and phase change arg[*T*(*ω*)], as shown in Figure 8. The error bars, indicating the measurement accuracy, correspond to the standard deviation of the average value obtained from data accumulations within multiple measurements.
